# Supplementary material for: Computational design of potent, broadly neutralizing anti-Nipah virus and Hendra virus miniproteins
Source: bioRxiv. 2026 Sep 17:2026.09.14.751546. Preprint. [Version 1] doi: 10.64898/2026.09.14.751546 (PMC13596370; doi:10.64898/2026.09.14.751546)
Supplement: Supplementary file 1 [file NIHPP2026.09.14.751546v1-supplement-1.pdf]

bioRxiv preprint doi: https://doi.org/10.64898/2026.09.14.751546; this version posted September 17, 2026. The copyright holder for this preprint (which was not certified by peer review) is the author/funder, who has granted bioRxiv a license to display the preprint in perpetuity. It is made available under a CC-BY 4.0 International license.

Supplementary Figures

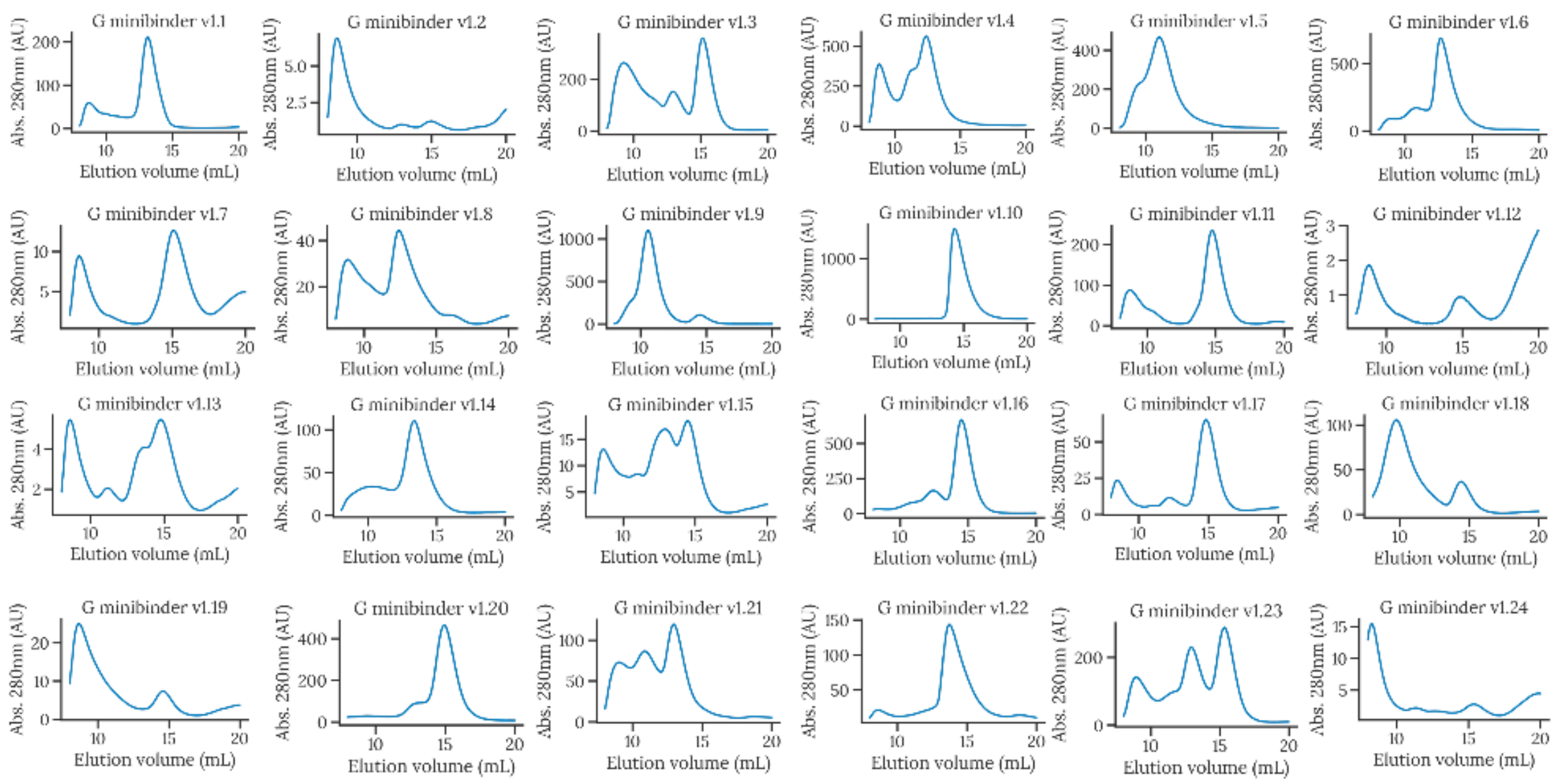

Supplementary Figure 1. SEC traces (S75 Increase 10/300 GL) curves of G minibinder v1 design series. G minibinder v1.10 identified via this screen became G minibinder v1 for subsequent testing and optimization.

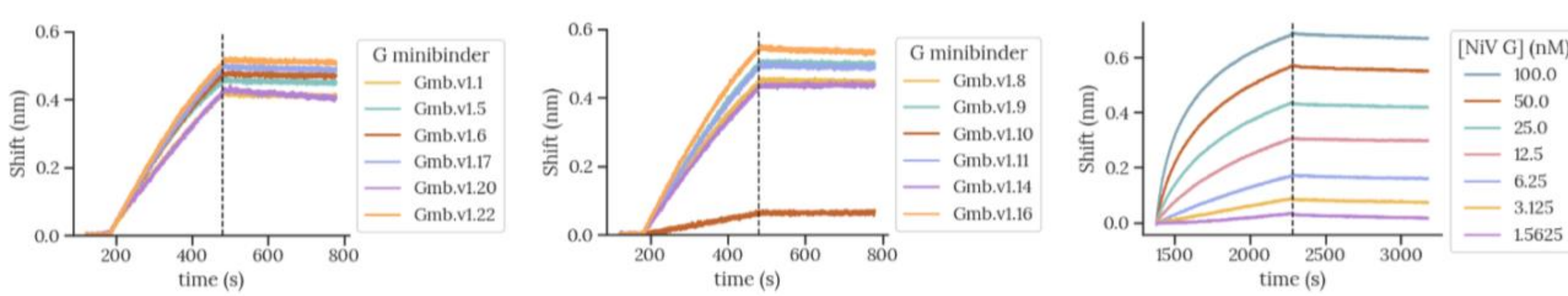

Supplementary Figure 2. Competition BLI **(left and middle)** and multicycle kinetics (**right**) of computationally designed G minibinder v1 designs. **Competition BLI** – Fc-tagged hEphrin-B2 was immobilized onto Protein A biosensors, baseline normalized in HBS-EP(+), and then dipped into 10 nM NiV G tetramer that was pre-incubated at room temperature

bioRxiv preprint doi: https://doi.org/10.64898/2026.09.14.751546; this version posted September 17, 2026. The copyright holder for this preprint (which was not certified by peer review) is the author/funder, who has granted bioRxiv a license to display the preprint in perpetuity. It is made available under a CC-BY 4.0 International license.

for 10 minutes with 1 μM SEC-purified G minibinder v1 designs (100x molar excess). Shown is the association phase (left of dashed line) and dissociation phase (right of dashed line) of NiV G tetramer in the presence of G minibinder. Minibinder alone traces (i.e. without NiV G in analyte) were subtracted from minbinder + NiV G traces to correct for nonspecific adsorption to biosensors due to high G minibinder concentrations – shown is the resultant trace. G minibinder v1.10 was the only design that promoted detectable reduction of NiV G binding signal by competition BLI. **Multicycle kinetics** – G minibinder v1.10 was immobilized via amine coupling to AR2G biosensors and NiV G tetramer was titrated down from 100 nM to 1.56 nM in a multicycle kinetics experiment to evaluate binding. Shown is the association phase (left of dashed line) and dissociation phase (right of dashed line) of NiV G tetramer.

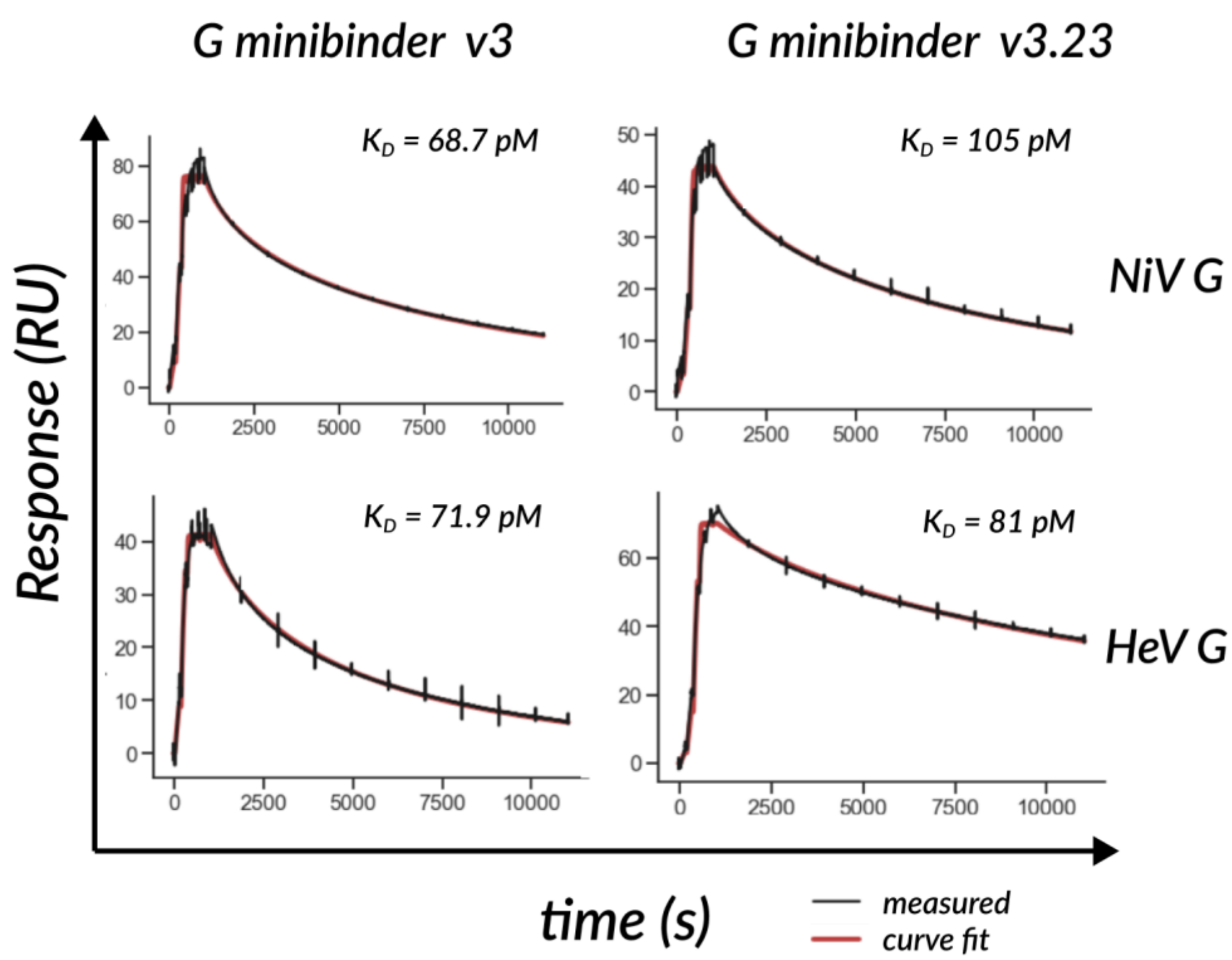

Supplementary Figure 3. Comparative binding kinetics of anti-G v3 and v3.23 minibinders with immobilized NiV G head (amine coupling via CM5 chip). Minibinders were titrated from 5 μM to 1.6 nM over 6 x 5-fold serial dilutions. Kinetic curves (black) fit with a 1:1 binding model model (color) to extract kinetic parameters for these monovalent proteins. Though being 86% sequence similar, the anti-G minibinder v3 bound NiV G with higher affinity to both NiV and HeV G and thus was elected for further downstream development.

bioRxiv preprint doi: https://doi.org/10.64898/2026.09.14.751546; this version posted September 17, 2026. The copyright holder for this preprint (which was not certified by peer review) is the author/funder, who has granted bioRxiv a license to display the preprint in perpetuity. It is made available under a CC-BY 4.0 International license.

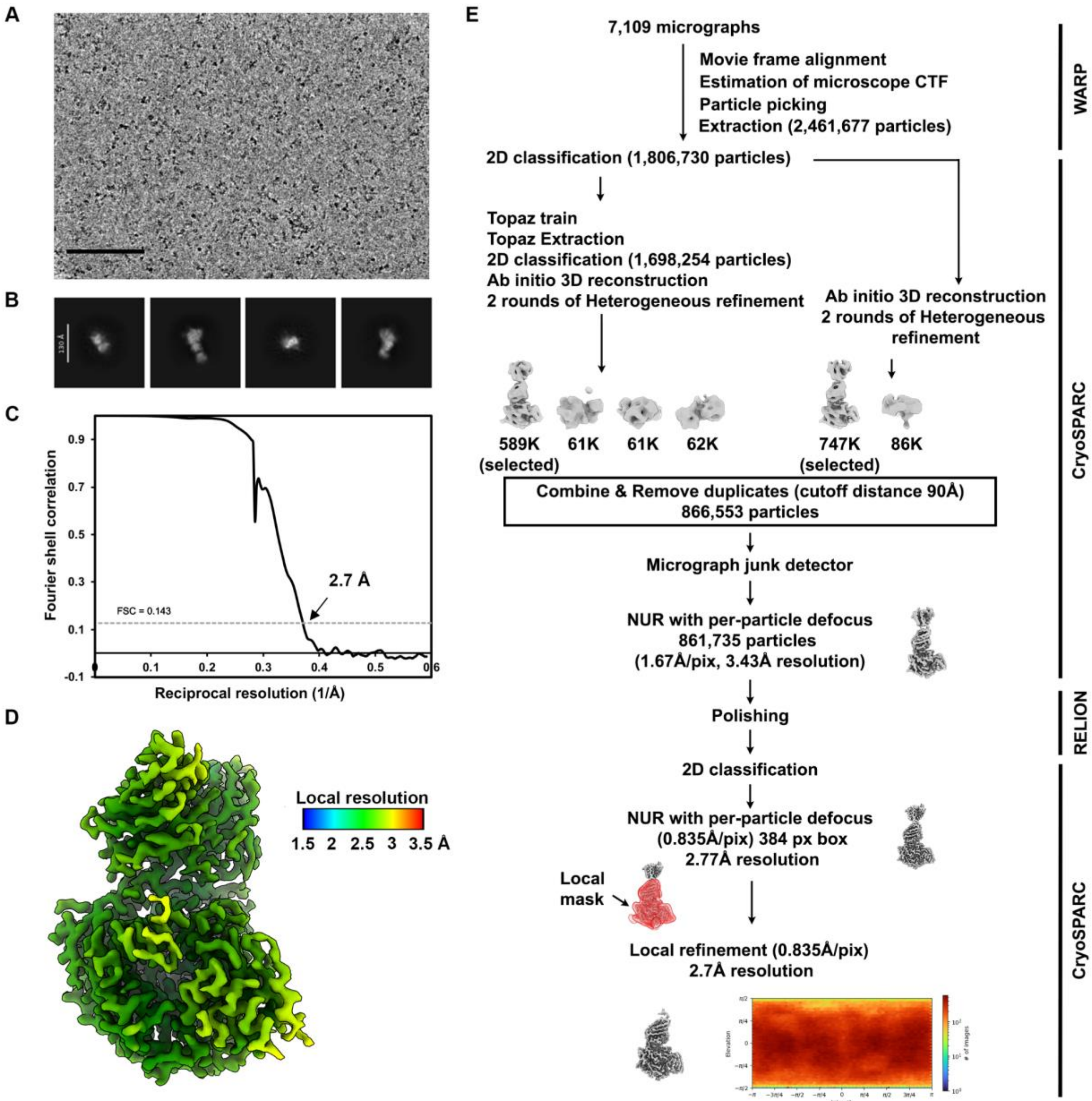

Supplementary Figure 4. Data collection and processing of the cryo-EM dataset of the vitrified NiV G head domain bound to the G minibinder v3 and HENV32 Fab.
(A-B) Representative electron micrograph and 2D class averages of the complex embedded in vitreous ice. Scale bars: 100 nm (A) and 130 Å (B). (C) Gold-standard Fourier shell correlation curve of the structure obtained by local refinement of the variable domains of the HENV32 Fab, G minibinder v3 and NiV G head. The 0.143 cutoff is indicated by a horizontal dashed line. (D) Local resolution estimation calculated using cryoSPARC and plotted on the corresponding sharpened reconstruction . (E) Data processing flowchart. CTF: contrast transfer function; NUR: non-uniform refinement. The angular distribution of particle images calculated using cryoSPARC is shown as a heat map.

bioRxiv preprint doi: https://doi.org/10.64898/2026.09.14.751546; this version posted September 17, 2026. The copyright holder for this preprint (which was not certified by peer review) is the author/funder, who has granted bioRxiv a license to display the preprint in perpetuity. It is made available under a CC-BY 4.0 International license.

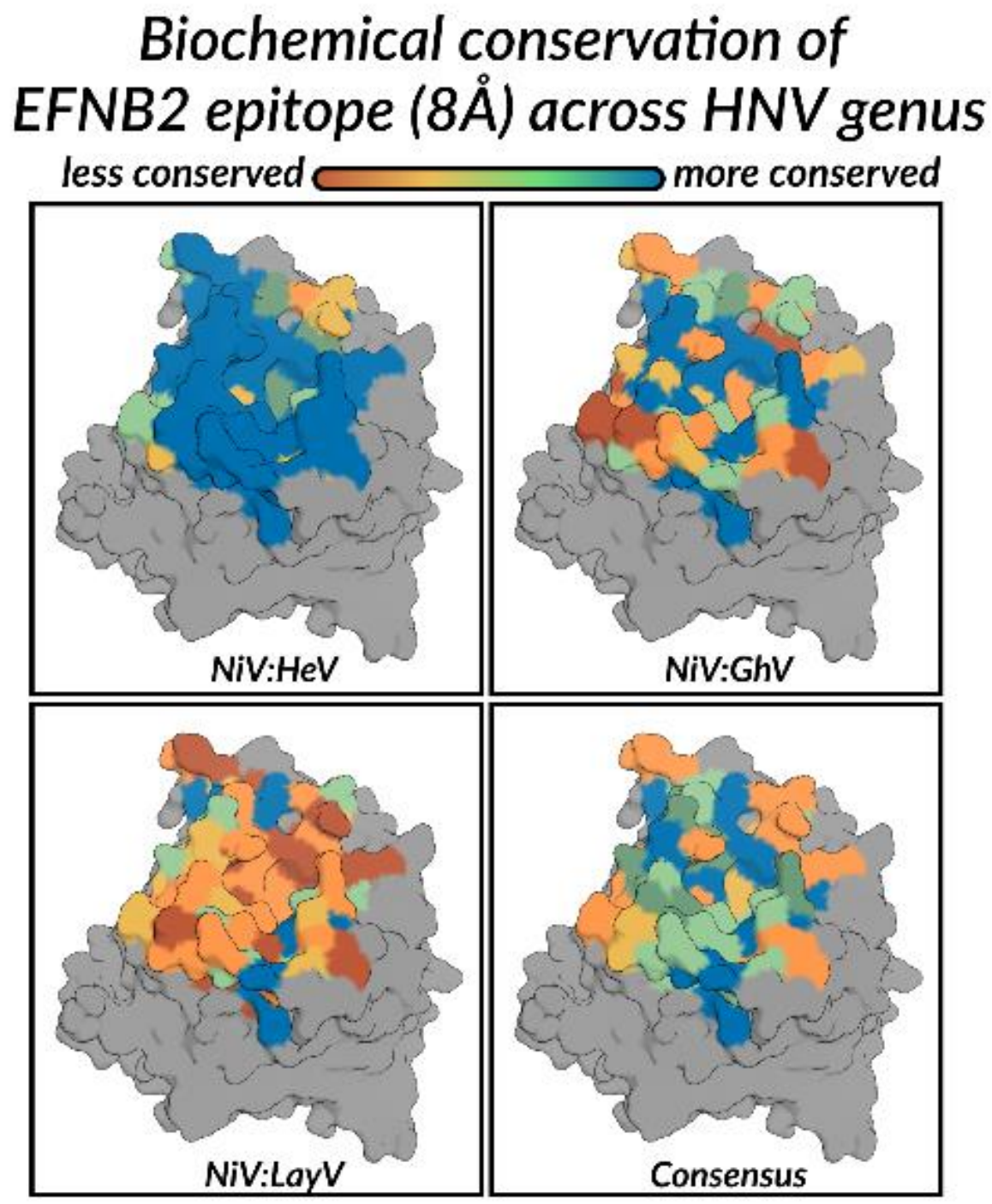

Supplementary Figure 5. Sequence conservation of EFNB2 binding site across selected henipaviruses and parahenipaviruses colored by BLOSUM-62-weighted biochemical similarity of residues of MAFFT-aligned sequences.

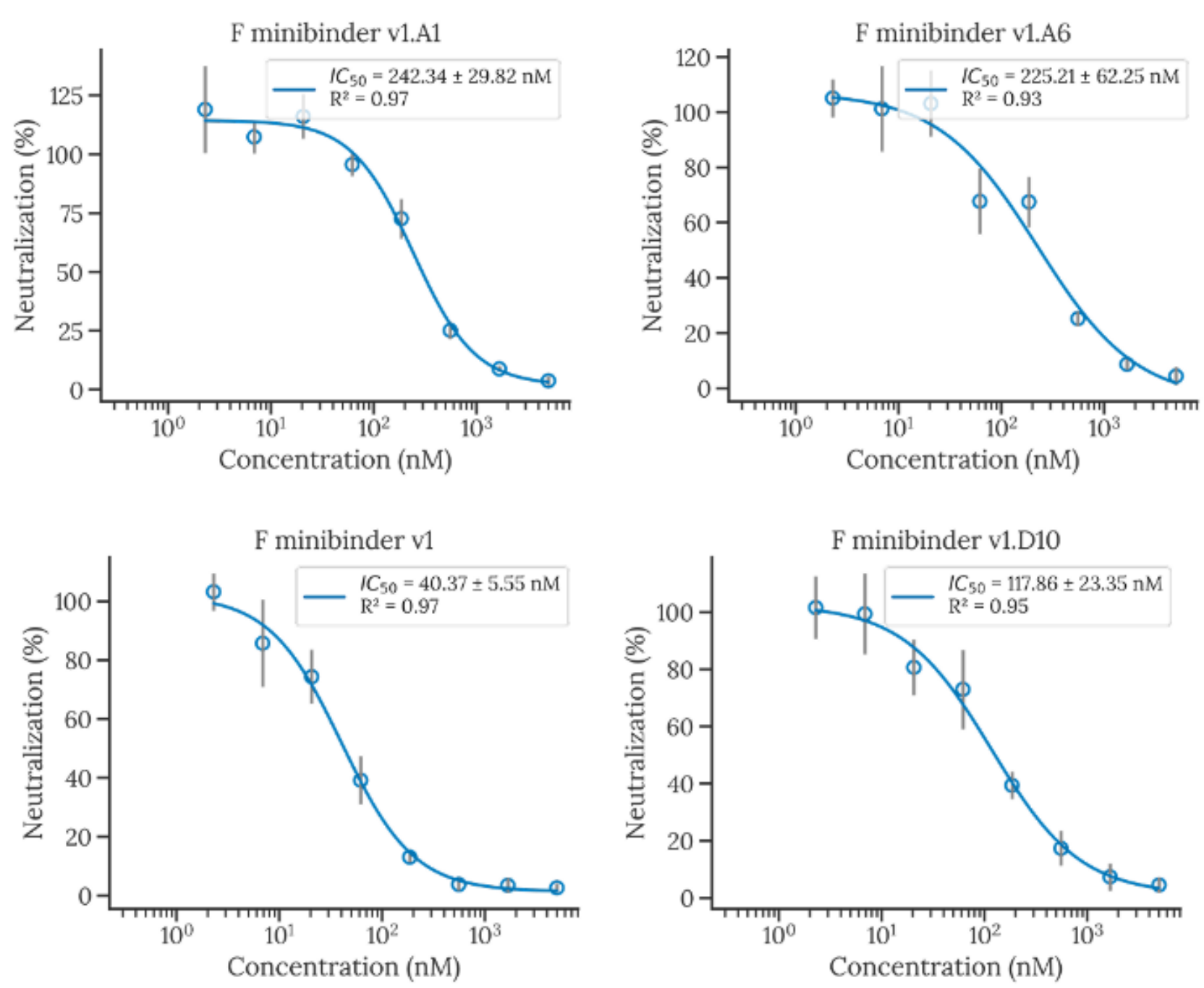

bioRxiv preprint doi: https://doi.org/10.64898/2026.09.14.751546; this version posted September 17, 2026. The copyright holder for this preprint (which was not certified by peer review) is the author/funder, who has granted bioRxiv a license to display the preprint in perpetuity. It is made available under a CC-BY 4.0 International license.

Supplementary Figure 6. Dose-response neutralization curves of F minibinder YSD hits with detectable binding affinity to NiV F. Neutralization experiments were run with HIV pseudotypes. Biological replicates (n=4) each composed of the mean of two technical duplicates at each concentration represented as a mean (dot) and standard deviation (vertical error bar). IC_{50} and standard error of the fit reported in the legend.

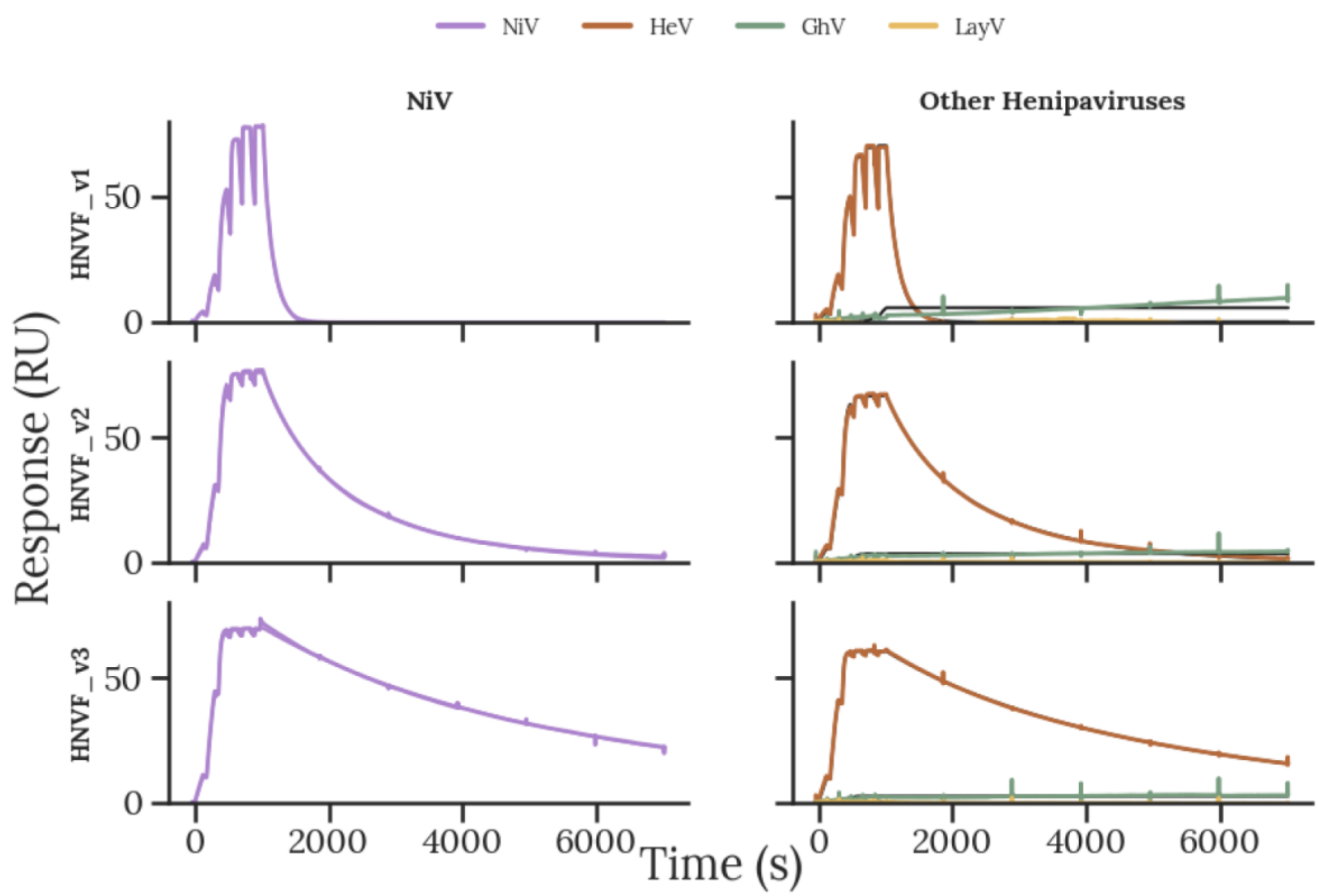

Supplementary Figure 7. SPR sensorgrams of F minibinder v3 binding to the immobilized prefusion F ectodomain trimers (amine coupling via CM5 chip). Minibinders were titrated from 5 µM to 1.6 nM over 6 x 5-fold serial dilutions. Kinetic curves (black) fit with a 1:1 binding model model (color) to extract kinetic parameters for these monovalent proteins. Binding was observed for NiV and HeV F, but not GhV and LayV F.

bioRxiv preprint doi: https://doi.org/10.64898/2026.09.14.751546; this version posted September 17, 2026. The copyright holder for this preprint (which was not certified by peer review) is the author/funder, who has granted bioRxiv a license to display the preprint in perpetuity. It is made available under a CC-BY 4.0 International license.

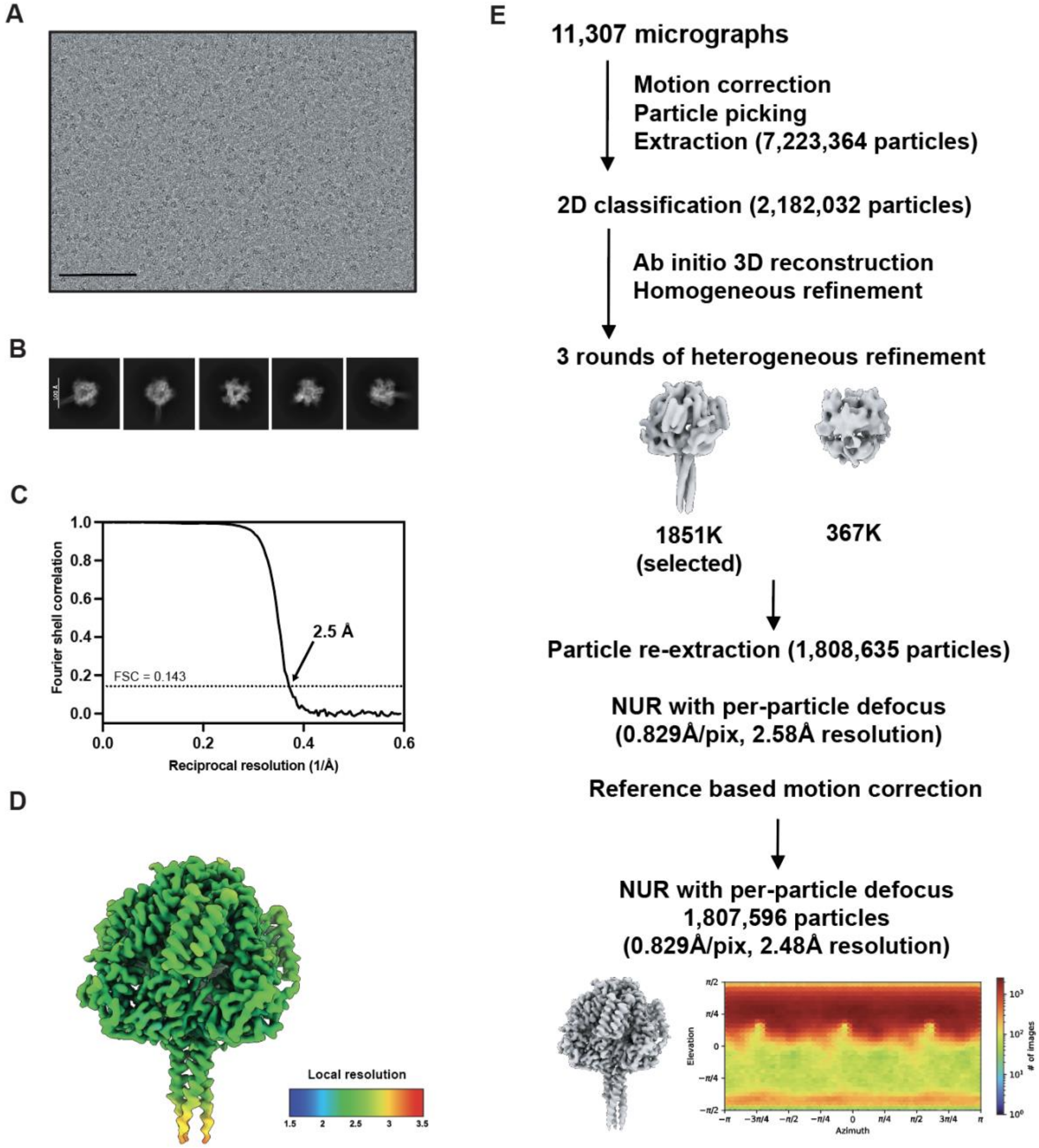

Supplementary Figure 8. Data collection and processing of the cryo-EM dataset of the vitrified prefusion NiV F bound to the F minibinder v3. (A-B) Representative electron micrograph and 2D class averages of the complex embedded in vitreous ice. Scale bars: 100 nm (A) and 100 Å (B). (C) Gold-standard Fourier shell correlation curve of the overall reconstruction. The 0.143 cutoff is indicated by a horizontal dashed line. (D) Local resolution estimation calculated using cryoSPARC and plotted on the sharpened reconstruction. (E) Data processing flowchart. NUR: non-uniform refinement. The angular distribution of particle images calculated using cryoSPARC is shown as a heat map.

bioRxiv preprint doi: https://doi.org/10.64898/2026.09.14.751546; this version posted September 17, 2026. The copyright holder for this preprint (which was not certified by peer review) is the author/funder, who has granted bioRxiv a license to display the preprint in perpetuity. It is made available under a CC-BY 4.0 International license.

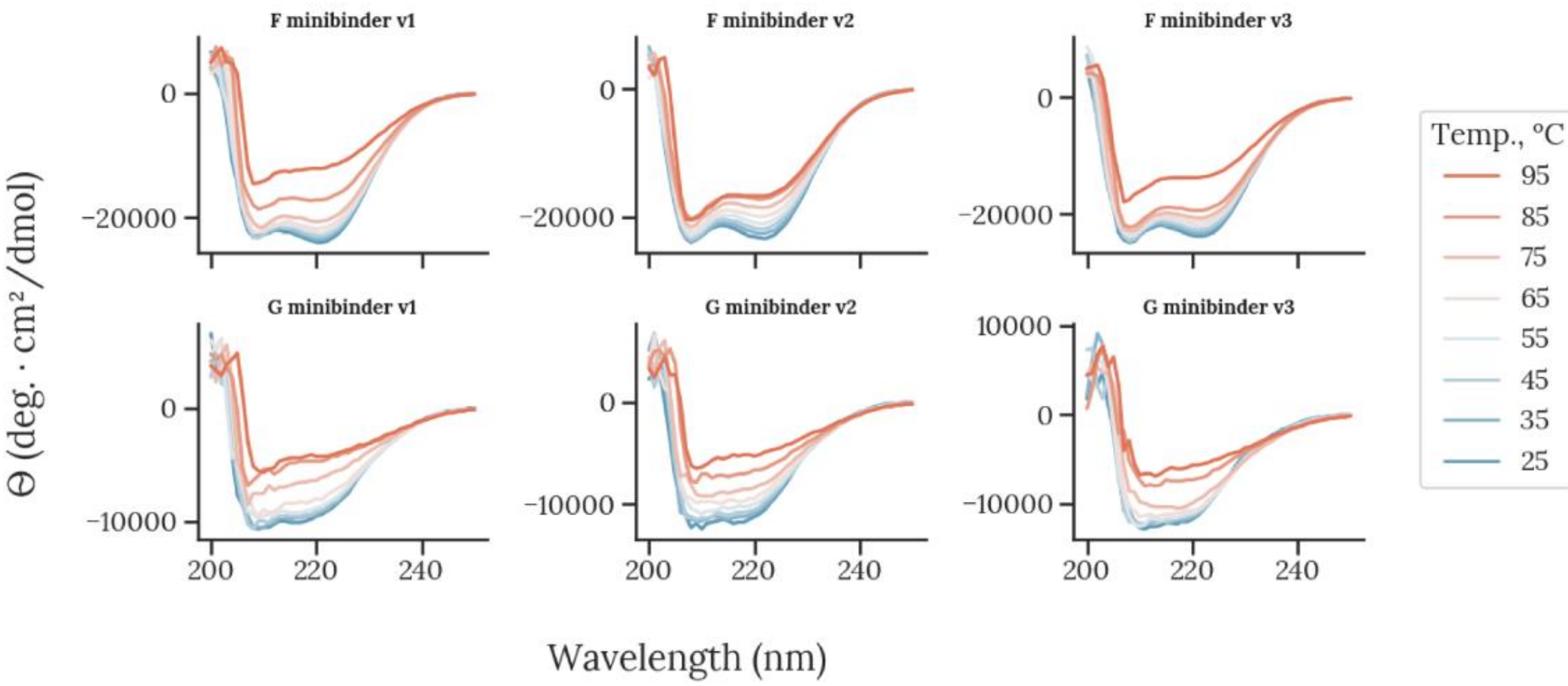

Supplementary Figure 9. Circular dichroism spectra of F & G minibinder v1-3 monomers at temperatures ranging from 25 - 95 °C indicate only partial loss of secondary structure at 85-95 °C for all monomers.

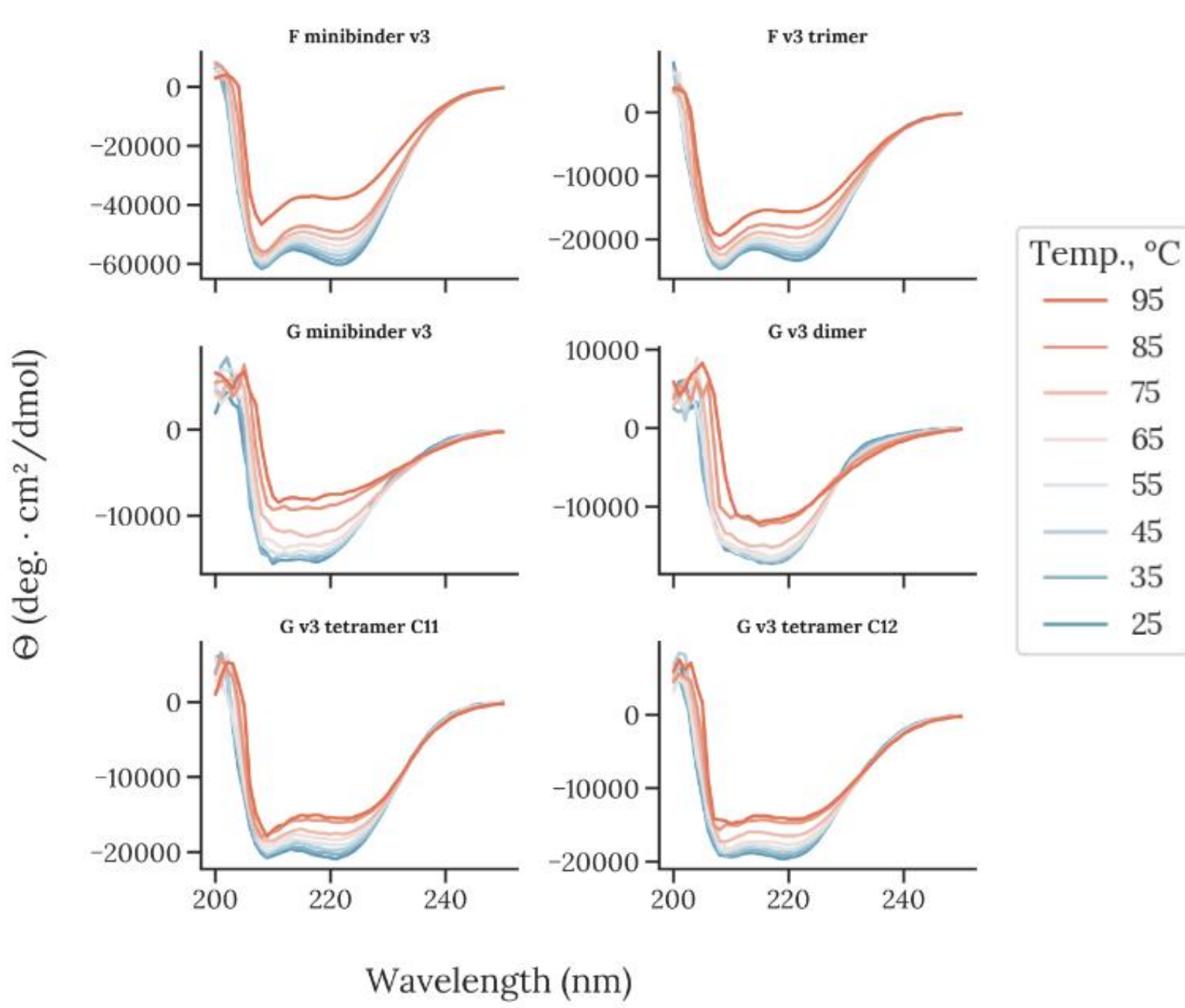

Supplementary Figure 10. Circular dichroism spectra of F & G minibinder v3 monomers

bioRxiv preprint doi: https://doi.org/10.64898/2026.09.14.751546; this version posted September 17, 2026. The copyright holder for this preprint (which was not certified by peer review) is the author/funder, who has granted bioRxiv a license to display the preprint in perpetuity. It is made available under a CC-BY 4.0 International license.

and oligomers at temperatures ranging from 25 - 95 °C indicate only partial loss of secondary structure at 85-95 °C for all constructs.

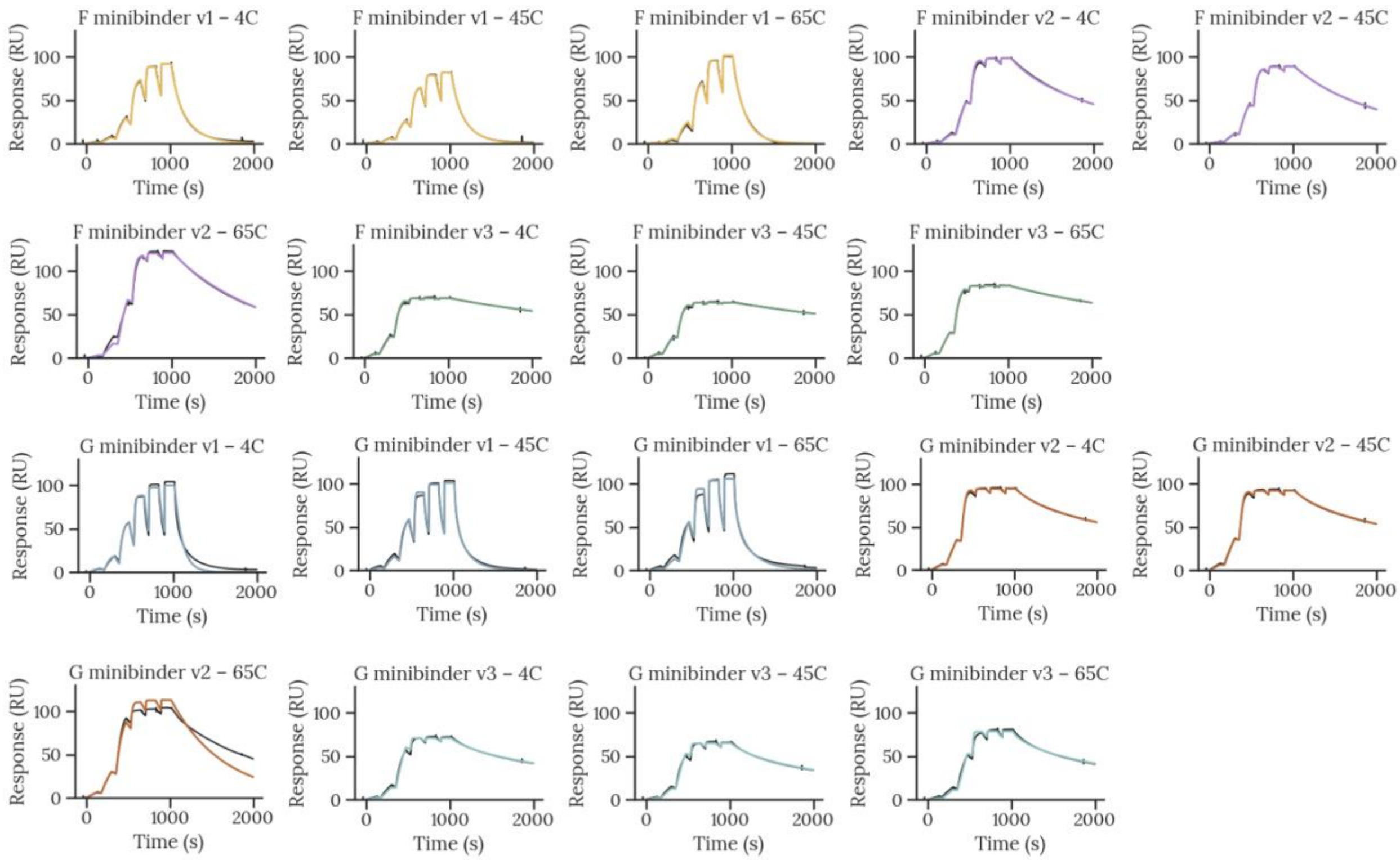

Supplementary Figure 11. SPR sensorgrams of F & G minibinder monomers incubated for 1 week at temperatures ranging from 4 - 65°C to immobilized NiV F or G (amine coupling via CM5 chip). Minibinders were titrated from 5 µM to 1.6 nM over 6 x 5-fold serial dilutions. Kinetic curves (black) fit with a 1:1 binding model model (color) to extract kinetic parameters for these monovalent proteins. Binding is preserved for all designs across the temperature range.

bioRxiv preprint doi: https://doi.org/10.64898/2026.09.14.751546; this version posted September 17, 2026. The copyright holder for this preprint (which was not certified by peer review) is the author/funder, who has granted bioRxiv a license to display the preprint in perpetuity. It is made available under a CC-BY 4.0 International license.

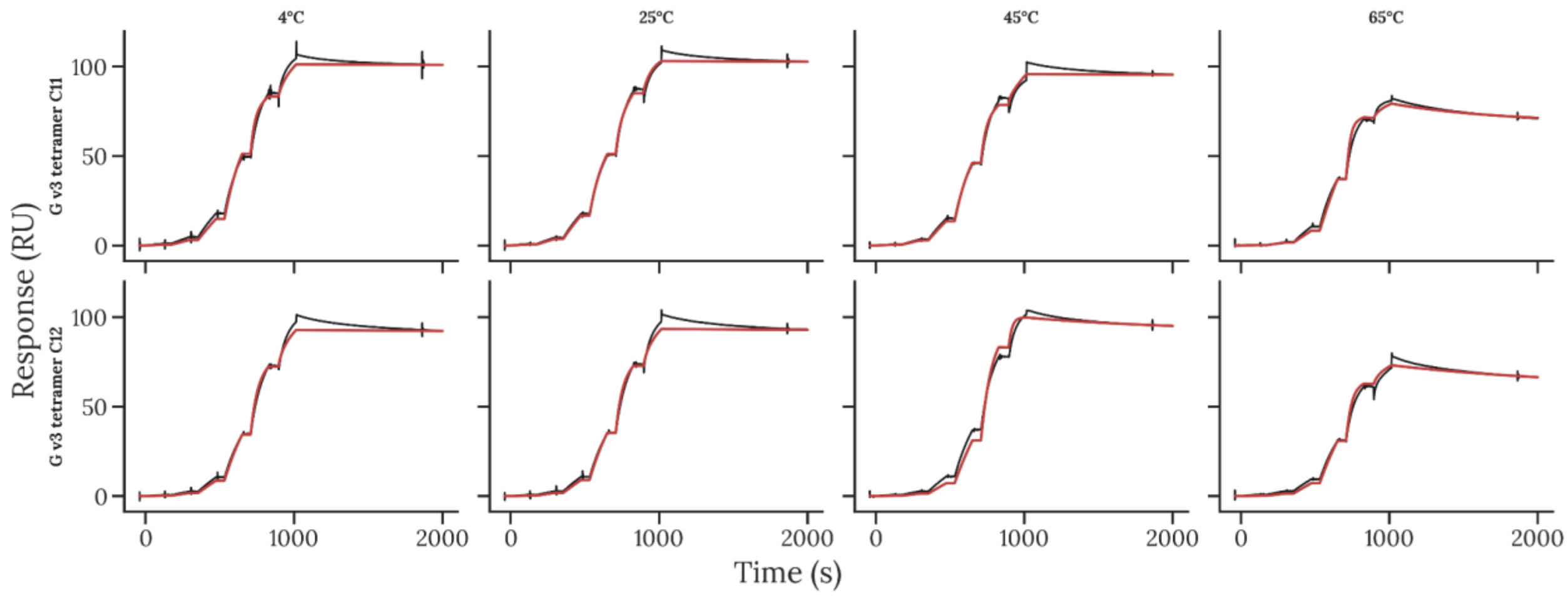

Supplementary Figure 12. SPR kinetic curves of G minibinder v3 tetramers C11 and C12 to immobilized NiV G tetramer (amine coupling via CM5 chip) after 1-week incubation in 1x PBS at 4, 25, 45, and 65°C. Loss of peak binding signal only observed at 65°C, suggesting loss from solution via aggregation or degradation. Minibinders were titrated from 5 μM to 1.6 nM over 6 x 5-fold serial dilutions. Kinetic curves (black) fit with a bivalent analyte model (red) to account for avidity due to multivalent analyte.

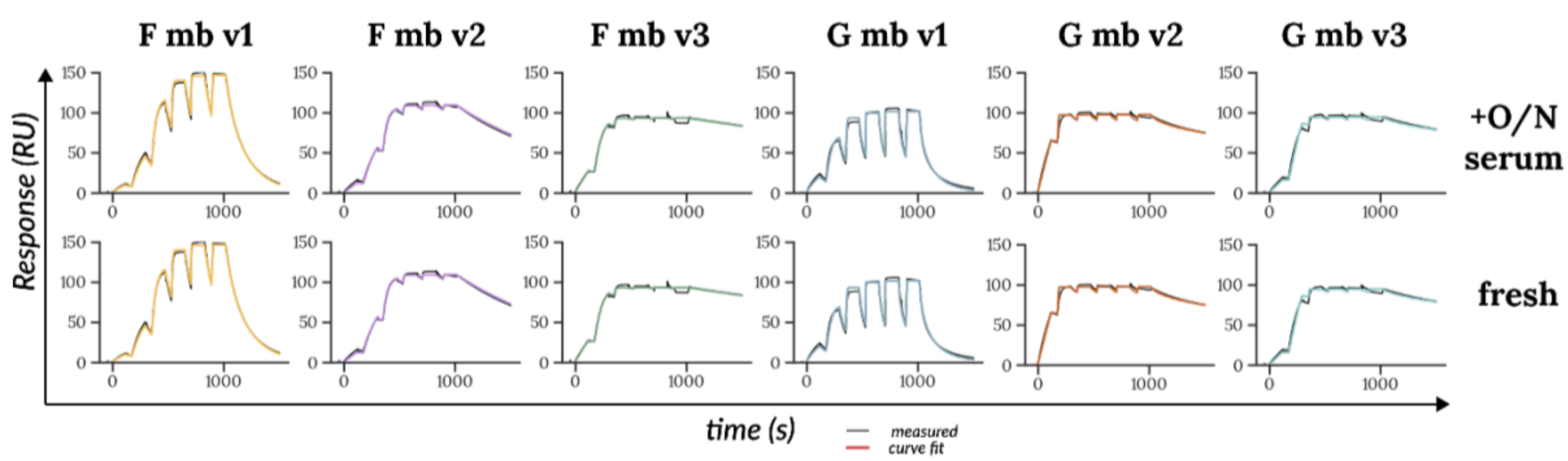

Supplementary Figure 13. SPR binding kinetics of F & G minibinder v1-3 monomers to immobilized prefusion NiV F ectodomain trimer (amine coupling via CM5 chip) after overnight incubation of minibinders in human serum at 37°C vs fresh addition to human serum prior to run. Minibinders were titrated from 5 μM to 1.6 nM over 6 x 5-fold serial dilutions. Kinetic curves (black) fit with a 1:1 binding model model (color) to extract kinetic parameters for these monovalent proteins.

bioRxiv preprint doi: https://doi.org/10.64898/2026.09.14.751546; this version posted September 17, 2026. The copyright holder for this preprint (which was not certified by peer review) is the author/funder, who has granted bioRxiv a license to display the preprint in perpetuity. It is made available under a CC-BY 4.0 International license.

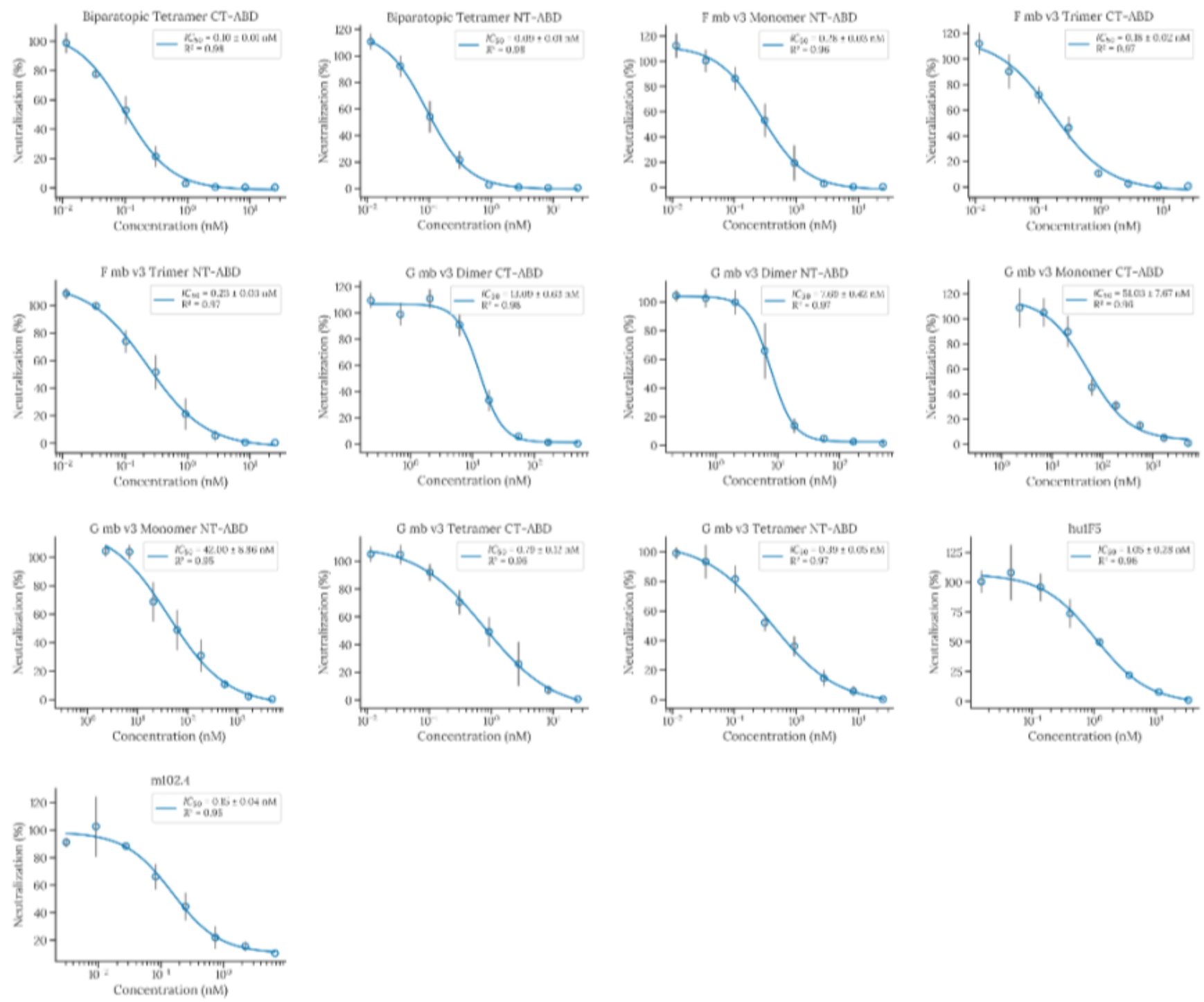

Supplementary Figure 14. Dose-response neutralization curves of F + G minibinder v3 monomers and oligomers compared with mAbs hu1F5 (anti-HNV F) and m102.4 (anti-HNV G). Technical replicates (n=4) at each concentration represented as a mean (dot) and standard deviation (vertical error bar). IC_{50} and standard error of the fit reported in the legend.

bioRxiv preprint doi: https://doi.org/10.64898/2026.09.14.751546; this version posted September 17, 2026. The copyright holder for this preprint (which was not certified by peer review) is the author/funder, who has granted bioRxiv a license to display the preprint in perpetuity. It is made available under a CC-BY 4.0 International license.

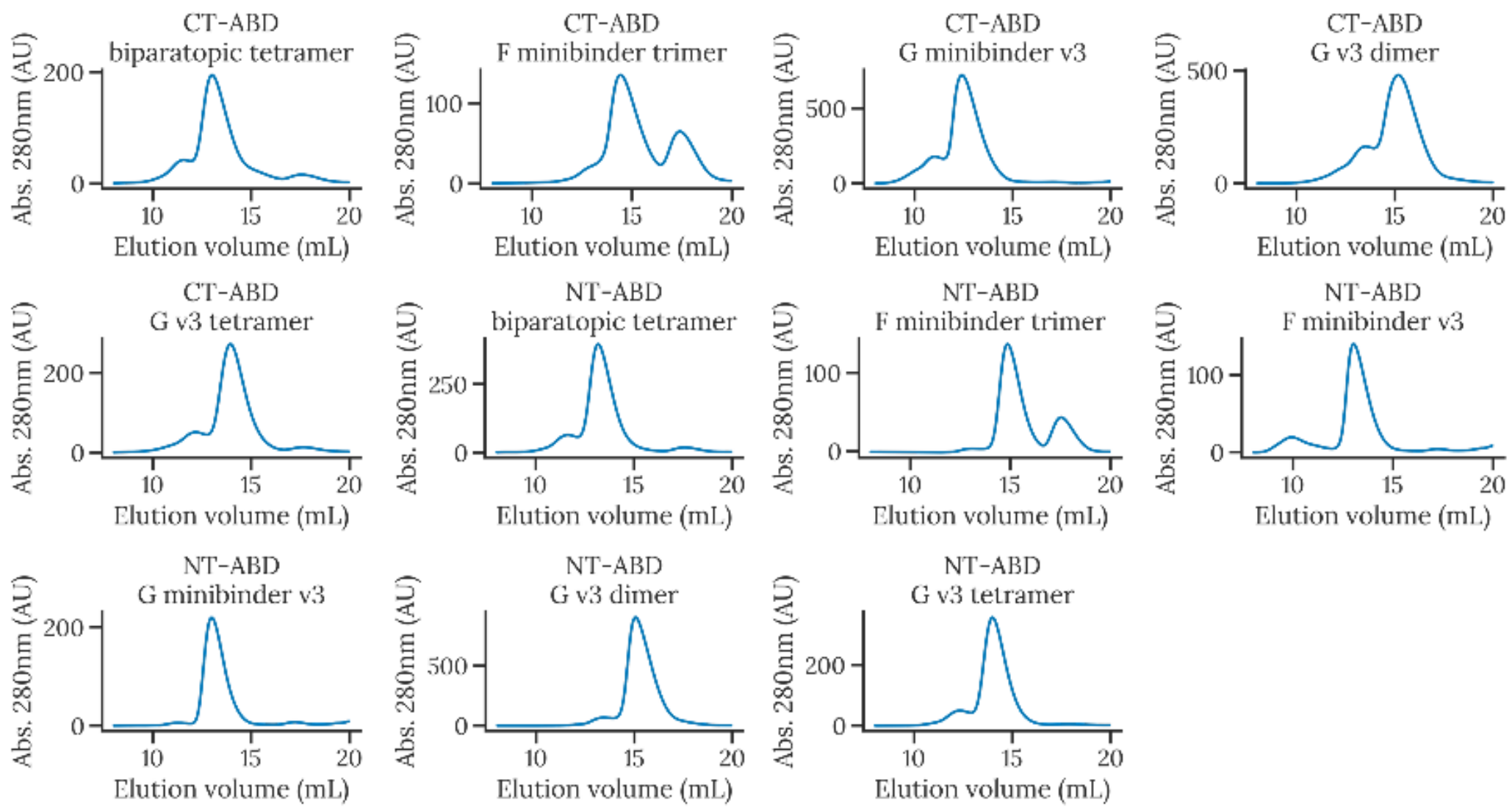

Supplementary Figure 15. Size exclusion chromatograms using Superdex S75 (for monomeric minibinders) or S200 (for oligomers) 10-300 GL Increase for v3 monomers and oligomers conjugated to albumin-binding domains recombinantly purified from E. *coli* and eluted in phosphate-buffered saline, pH 7.4.

Supplementary Table 1. SPR kinetics and NiV F & G-pseudotyped HIV IC_{50} values of F minibinder v1 designs.

**Design name**	**NiV F K_D (M)**	**NiV F k_a (1/Ms)**	**NiV F k_d (1/s)**	**HeV F K_D (M)**	**HeV F k_a (1/Ms)**	**HeV F k_d (1/s)**	**IC_{50} (nM)**	**IC_{50} std. dev. (nM)**	**R^2**
F mb v1.A1	1.21E-07	217000	0.0262	1.04E-07	260000	0.0272	242.3373	29.8159	0.966217
F mb v1.A6	1.19E-07	599000	0.0712	7.68E-08	618000	0.0474	225.2061	62.24712	0.931804
F mb	1.55E-	462000	0.007	4.34E	45800	0.019	117.8	23.35119	0.949

bioRxiv preprint doi: https://doi.org/10.64898/2026.09.14.751546; this version posted September 17, 2026. The copyright holder for this preprint (which was not certified by peer review) is the author/funder, who has granted bioRxiv a license to display the preprint in perpetuity. It is made available under a CC-BY 4.0 International license.

v1.D10	08		16	-09	00	9	649		69
F mb v1.A7 **(v1)**	7.43E-08	604000	0.0449	3.5E-08	738000	0.0258	40.37356	5.546531	0.968555

Supplementary Table 2. IC_{50} values of ABD-conjugated F and G minibinder v3 monomers and oligomers tested in NiV F & G-pseudotyped HIV assay.

Design name	**IC_{50}**	**IC_{50} std. dev.**	**R^2**
G mb v3 Monomer NT-ABD	41.99717	8.863075	0.951728
F mb v3 Trimer CT-ABD	0.175589	0.021899	0.968035
G mb v3 Monomer CT-ABD	51.03011	7.674627	0.955398
Biparatopic Tetramer CT-ABD	0.096955	0.008909	0.981219
G mb v3 Dimer CT-ABD	13.08776	0.633395	0.980098
F mb v3 Monomer NT-ABD	0.279482	0.02757	0.964669
G mb v3 Tetramer NT-ABD	0.389201	0.050227	0.969691
G mb v3 Tetramer CT-ABD	0.786113	0.121969	0.960881
G mb v3 Dimer NT-ABD	7.686403	0.424577	0.971988
Biparatopic Tetramer NT-ABD	0.09189	0.007162	0.981786
F mb v3 Trimer NT-ABD	0.228445	0.025805	0.97351
m102.4	0.152094	0.040877	0.950154
